# Letter from J. T. Newman, M. D.

**Published:** 1868-01-15

**Authors:** James T. Newman

**Affiliations:** Chicago


CHICAGO, *December*, 1867.

*Editor Chicago Medical Journal:*

B. A., a mulatto girl, came to my office, to be examined. Stout, heavy build, and about 22 years of age.

She stated that she suffered excruciating pains through the bowels, down the inner part of the thighs, to the foot. When one of her paroxysms would come on, which occurred every few minutes, there would be, almost simultaneously with it, a congestion of the saphenous veins; they would become so turgid that one might easily be led to believe it to be phlebitis. This would last probably from three to five minutes, and then disappear almost as suddenly as it had appeared. During the congestion of these veins, she complained of great pain, burning of the vulva and clitoris. This condition of things, I learned had been the case for six or eight months. The nates were so tender that she could not sit, with any degree of comfort, even on a soft seat; and then, only for a few minutes. The menses had been suppressed for eight months, during which time the bowels had been constipated, so much so that there was no action of them, except by artificial means. Leucorrhœa had been very profuse, with headache and loss of appetite. In micturition, the pain was almost unendurable — represented by her as if there was something obstructing the flow of urine, and feeling as if the parts were on fire. She said she had been under the treatment of several physicians, but they had given her neither relief nor satisfaction, as to what was the matter with her, or if she could be relieved.

Upon an examination, I found the parts much inflamed, tumefied and hot; the clitoris from two to two and a half inches in length, and quite as large as a man's thumb, very red, dry, hot, and obstructing the entrance to the vagina so that it was difficult to introduce even the finger. I tried several times to effect an introduction of the speculum; but the parts were so tender, and the clitoris so long and large, that it was impossible.

She was directed to use a wash three times a day, composed of *Cupri sulphat.* ʒj., to *Aquæ puræ*, Oj.; then dry the parts well with a soft towel or cloth, and dust with powdered starch. For the constipation and suppression, she was directed *Aloes soc.*, grs. xx.; extract *Nux vomica*, grs. v.; make in pills, xx. S. one morning, noon and night; and go to stool every morning, at a certain hour, whether there was a desire to defecate or not.

I have never seen the case since but once, and then only to meet her on the street; but have learned, through others, that the treatment succeeded well. She commenced improving from the time she first used the wash; the pains all left; there was a rapid restoration of the clitoris to its normal condition and size; the bowels soon became regular, and the menstrual flow was reëstablished; also her general health.

JAMES T. NEWMAN, M.D.

